# Position paper of the EACVI and EANM on artificial intelligence applications in multimodality cardiovascular imaging using SPECT/CT, PET/CT, and cardiac CT

**DOI:** 10.1007/s00259-021-05341-z

**Published:** 2021-04-17

**Authors:** Riemer H. J. A. Slart, Michelle C. Williams, Luis Eduardo Juarez-Orozco, Christoph Rischpler, Marc R. Dweck, Andor W. J. M. Glaudemans, Alessia Gimelli, Panagiotis Georgoulias, Olivier Gheysens, Oliver Gaemperli, Gilbert Habib, Roland Hustinx, Bernard Cosyns, Hein J. Verberne, Fabien Hyafil, Paola A. Erba, Mark Lubberink, Piotr Slomka, Ivana Išgum, Dimitris Visvikis, Márton Kolossváry, Antti Saraste

**Affiliations:** 1 Medical Imaging Centre, Department of Nuclear Medicine and Molecular Imaging, University of Groningen, University Medical Center Groningen, Hanzeplein 1, PO 9700 RB Groningen, The Netherlands; 2 Faculty of Science and Technology Biomedical, Photonic Imaging, University of Twente, Enschede, The Netherlands; 3 British Heart Foundation Centre for Cardiovascular Science, University of Edinburgh, Edinburgh, UK; 4 Edinburgh Imaging facility QMRI, Edinburgh, UK; 5 Department of Cardiology, Division Heart & Lungs, University Medical Center Utrecht, Utrecht University, Utrecht, the Netherlands; 6 University Medical Center Groningen, University of Groningen, Groningen, The Netherlands; 7 Department of Nuclear Medicine, University Hospital Essen, University of Duisburg-Essen, Essen, Germany; 8 Fondazione Toscana G. Monasterio, Pisa, Italy; 9 Department of Nuclear Medicine, Faculty of Medicine, University of Thessaly, University Hospital of Larissa, Larissa, Greece; 10 Department of Nuclear Medicine, Cliniques Universitaires Saint-Luc and Institute of Clinical and Experimental Research (IREC), Université catholique de Louvain (UCLouvain), Brussels, Belgium; 11 HeartClinic, Hirslanden Hospital Zurich, Zurich, Switzerland; 12 APHM, Cardiology Department, La Timone Hospital, Marseille, France; 13 IRD, APHM, MEPHI, IHU-Méditerranée Infection, Aix Marseille Université, Marseille, France; 14 Division of Nuclear Medicine and Oncological Imaging, Department of Medical Physics, ULiège, Liège, Belgium; 15 Department of Cardiology, Centrum voor Hart en Vaatziekten, Universitair Ziekenhuis Brussel, 101 Laarbeeklaan, 1090 Brussels, Belgium; 16 Department of Radiology and Nuclear Medicine, Amsterdam UMC, location AMC, University of Amsterdam, Amsterdam, The Netherlands; 17 Department of Nuclear Medicine, DMU IMAGINA, Georges-Pompidou European Hospital, Assistance Publique - Hôpitaux de Paris, F-75015 Paris, France; 18 University of Paris, PARCC, INSERM, F-75006 Paris, France; 19 Department of Nuclear Medicine (P.A.E.), University of Pisa, Pisa, Italy; 20 Department of Translational Research and New Technology in Medicine (P.A.E.), University of Pisa, Pisa, Italy; 21 Department of Surgical Sciences/Radiology, Uppsala University, Uppsala, Sweden; 22 Medical Physics, Uppsala University Hospital, Uppsala, Sweden; 23 Department of Imaging, Medicine, and Biomedical Sciences, Cedars-Sinai Medical Center, Los Angeles, CA USA; 24 Department of Biomedical Engineering and Physics, Amsterdam UMC - location AMC, University of Amsterdam, 1105 Amsterdam, AZ Netherlands; 25 INSERM, LaTIM, UMR 1101, Univ Brest, Brest, France; 26 MTA-SE Cardiovascular Imaging Research Group, Heart and Vascular Center, Semmelweis University, 68 Városmajor Street, Budapest, Hungary; 27 Turku PET Centre, Turku University Hospital, University of Turku, Turku, Finland; 28 Heart Center, Turku University Hospital, Turku, Finland

**Keywords:** Position paper, Machine learning, Deep learning, Cardiovascular, Multimodality imaging

## Abstract

In daily clinical practice, clinicians integrate available data to ascertain the diagnostic and prognostic probability of a disease or clinical outcome for their patients. For patients with suspected or known cardiovascular disease, several anatomical and functional imaging techniques are commonly performed to aid this endeavor, including coronary computed tomography angiography (CCTA) and nuclear cardiology imaging. Continuous improvement in positron emission tomography (PET), single-photon emission computed tomography (SPECT), and CT hardware and software has resulted in improved diagnostic performance and wide implementation of these imaging techniques in daily clinical practice. However, the human ability to interpret, quantify, and integrate these data sets is limited. The identification of novel markers and application of machine learning (ML) algorithms, including deep learning (DL) to cardiovascular imaging techniques will further improve diagnosis and prognostication for patients with cardiovascular diseases. The goal of this position paper of the European Association of Nuclear Medicine (EANM) and the European Association of Cardiovascular Imaging (EACVI) is to provide an overview of the general concepts behind modern machine learning-based artificial intelligence, highlights currently prefered methods, practices, and computational models, and proposes new strategies to support the clinical application of ML in the field of cardiovascular imaging using nuclear cardiology (hybrid) and CT techniques.

## Introduction

In daily clinical practice, clinicians integrate available data to ascertain the diagnostic and prognostic probability of a disease or clinical outcome for their patients. For patients with suspected or known cardiovascular disease, several anatomical and functional imaging techniques are commonly performed to aid this endeavor, including coronary computed tomography angiography (CCTA) and nuclear cardiology imaging. Continuous improvement in positron emission tomography (PET), single-photon emission computed tomography (SPECT), and CT hardware and software has resulted in improved diagnostic performance and wide implementation of these imaging techniques in the daily clinical practice. However, the human ability to interpret, quantify, and integrate these data sets are limited. The identification of novel markers and application of machine learning (ML) algorithms, including deep learning (DL) to cardiovascular imaging techniques will further improve diagnosis and prognostication for patients with cardiovascular diseases [1].

## Goal

This position paper of the European Association of Nuclear Medicine (EANM) and the European Association of Cardiovascular Imaging (EACVI) provides an overview of the general concepts behind modern machine learning-based artificial intelligence; highlights currently prefered methods, practices, and computational models; and proposes new strategies to support the clinical application of ML in the field of cardiovascular imaging using nuclear cardiology (hybrid) and CT techniques.

## Background

**Fig. 1:**
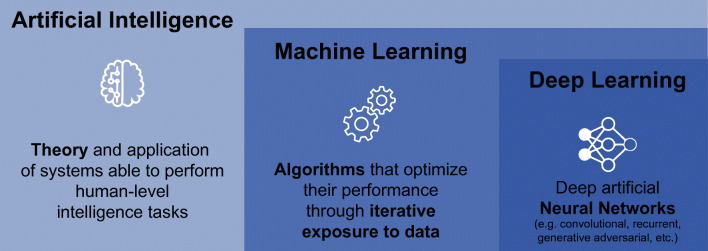
Conceptual Framework. Modified from Juarez-Orozco et al. [37]

Artificial intelligence (AI) is a general term used to describe computational processes that mimic or surpass human intelligence [2]. For a system to be intelligent, it needs to think and act humanly (we cannot distinguish it from other humans’ thoughts and actions) and rationally (it decides and responds optimally under all circumstances) [3]. Similar to the way humans learn, AI algorithms require many training examples to accomplish a task with confidence. This has led to a more general formalization of AI, which defines intelligent algorithms as ones that increase their performance in a given task (evaluated by some performance metric) proportional to the amount of experience they possess [4] (Fig. 1).

At the core of AI processes is machine learning (ML), an umbrella term for statistical and analytical techniques that accomplish a classification or prediction task without being explicitly programmed for that purpose. ML approaches in imaging require expert-engineered image characteristics that are important for the classification or regression task at hand. The extracted features are subsequently used as input to a statistical classifier that performs the task.

**Fig. 2:**
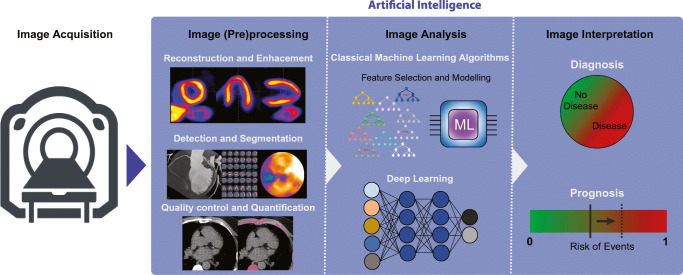
Artificial intelligence assisted image analysis. Artificial intelligence is currently fueled by machine learning algorithms, which can be roughly classified into: classical machine learning models and deep learning models. These can be used in a variety of imaging tasks, including pre-processing, image analysis, and image interpretation. ML machine learning

Statistical ML analysis can be used to assess many features obtained from medical images. There are over a hundred different statistical classifiers in ML. Some can be seen as evolutions of regression techniques and some are based on concepts mimicking the way humans think (i.e., decision trees). A common feature of these techniques is that they are capable of modelling complex non-linear relationships, allowing the model to better fit the data. This means that the data need to be diverse and representative to minimize the chance of overfitting the model and losing generalizability. In medical imaging, we can use the pixel values themselves as inputs for ML, or we can generate features from images that aim to quantify different aspects of the image (Fig. 2). The process of extracting a very large set of quantitative features mostly describing the shape and texture is called radiomics. This generative technique aims to extract a very large set of quantitative features from medical images that describe the texture and geometry of the image to create big-data databases for ML analysis [5].

Deep learning (DL) is a subset of ML algorithms, which uses artificial neural networks to filter input data through a series of layers. The main property of DL is that the models automatically perform a search and selection of the most relevant features learn, and learn directly from the data through an optimization process. In tasks involving images, convolutional neural networks (CNN) are often used. CNNs directly process images through a series of connected layers in order to deliver image classification and regression tasks.

In ML, the process of *learning* can be undertaken in several distinct ways. The two most used learning approaches are supervised and unsupervised learning. In *supervised learning*, labelled data is available to the ML or DL algorithm during the training process, such as the presence of an imaging finding (e.g., coronary artery calcification), disease (e.g., amyloidosis), or cardiovascular outcome (e.g., myocardial infarction) is provided as the ground truth against which the algorithm’s output is compared. On the other hand, *unsupervised learning* does not require labelled data during training. Instead it tries to identify patterns and clusters itself within data (e.g., new subgroups of patients with heart failure). *Reinforcement learning* is a different technique where the ML agent learns from its environment and experiences based on feedback from rewards and punishment, such as those used to train AlphaGo to play the board game Go and the self-driving algorithm of Tesla. Obtaining labelled data in medical image analysis can be time-consuming and expensive, and a variety of methods have been developed to address this issue. *Transfer learning* involves the use of pre-trained models (e.g., DL network architectures), which can be applied to a new classification task. This enables the transfer of knowledge from one domain with available/abundant labelled data (e.g., facial recognition) to another domain (e.g., medical imaging), thereby reducing the requirements on the amount of training data required in the new domain. *Weak supervision* involves the use of noisy, limited, or imprecisely labelled data for supervised learning. Finally, *Semi-supervised learning* involves a combined approach involving a small number of labelled data and a larger volume of unlabelled data to reduce the need for labelled training data. Despite the limitations of the previous two methods, accurate models can be created if there is a large volume of data.

## General aspects of AI in cardiovascular imaging

There are many tasks in cardiovascular imaging that may benefit from ML. First, general potential applications of AI in cardiovascular imaging are discussed, followed by imaging modality specific AI application (Fig. 2).

### Image acquisition and reconstruction

In the past decade, image reconstruction algorithms have evolved in parallel with imaging hardware allowing for dramatic improvements in image qualitative and quantitative accuracy, resulting in a reduction in acquisition times and/or radiation exposure. In order to achieve such improved performance, different data corrections have been accurately modelled and incorporated [6, 7], within iterative reconstruction algorithms, which may impact their computational time efficiency in cardiovascular imaging [8–11]. Although current iterative algorithms used in clinical practice provide excellent image quality, there are issues concerning the variability in convergence rate as a function of activity concentrations in the different tissues of interest. There is clearly room for further improvements, particularly within the context of low radiation exposure and parametric imaging. Image reconstruction using DL methods is not as widely explored as in other areas of cardiovascular imaging, such as image segmentation and classification tasks. Most current implementations are embedded in classical iterative reconstruction algorithms (e.g., denoising of successive image estimations in each iteration) [12]. In addition, ML analysis may be performed on the raw data output from scanners, bypassing the image reconstruction step altogether [13]. A few direct DL reconstruction approaches have been proposed, allowing the creation of reconstructed images from raw data [14–17]. There is also a substantial body of work concerning the use of DL methods for data corrections (e.g., attenuation, scatter) during the reconstruction process [15, 18] and image post-processing algorithms (denoising, super-resolution, artefact removal) [19–21]. DL methods can also be used to reduce motion artifact in cardiac imaging, more frequently used at present in MRI [22]. Although these first implementations demonstrate the feasibility of DL approaches for tomographic reconstruction and associated data corrections, there is a clear lack of comparative studies, limiting the ability to assess their robustness and quantitative accuracy in different clinical scenarios compared to current state of the art iterative reconstruction frameworks [23].

### Image segmentation

Image segmentation is a process in which an image is subdivided into anatomically meaningful parts/segments [24], and it represents one of the most “mature” applications of DL in medical imaging in general, but also specifically in cardiovascular imaging [25]. U-NET is a good example of a widely used segmentation network. U-NET in its 2D and 3D implementation is the current state-of-the-art segmentation algorithm used for a variety of imaging modalities and clinical applications and has shown in different recent segmentation challenges its strong potential to become the state-of-the-art in medical image segmentation [26].

### Image registration

Accurate registration of images is important for hybrid and multimodality imaging such as PET/CT and PET/MR, and some investigators have demonstrated the potential for improvements in image registration accuracy for anatomical and functional image deformations [26, 27]. Moreover, this approach can help with correction of both physiological (cardiac, respiratory) and involuntary motion. However, clearly more work is needed in order to demonstrate the potential interest of DL-based approaches relative to current state-of-the-art.

### Image analysis, precision phenotyping, disease reclassification, and risk stratification

Recently, there has been increasing interest in the use of imaging (bio) markers in medical imaging. The additive value of radiomics and ML (especially through DL) will on the one hand greatly increase the amount of information accessible from images, and on the other, facilitate its integration in order to amplify our insights into cardiovascular pathological states [28, 29]. This new information will allow for precision phenotyping and a more accurate classification of diseases, potentially changing our current taxonomies [30]. The large majority of radiomics work in medical imaging for cardiac applications has been in the field of MR and CT [31], with only little work as yet in the field of nuclear medical imaging [32, 33].

The ultimate goal of imaging is to better understand the clinical status of the patient and assess the risk of subsequent cardiovascular events. AI techniques can utilize information from multiple sources and have the potential to make decisions considering all available information. This paves the road to revolutionize medical care as it has the potential to provide more accurate and more individual risk prediction and thereby may help in better medication prescription and the use of more invasive (therapeutic) interventions [29], although there is a need for a more rigorous multi-center validation of the developed predictive and prognostic models.

## AI in cardiovascular SPECT (CT) and PET (CT)

AI is rapidly permeating into nuclear cardiac imaging taking advantage of the large existing and standardized imaging database available in this field [34, 35]. Given that automated imaging processing and analyses have been used in nuclear imaging for 20 years, it is likely that the benefits of implementing AI approaches could be first evaluated in these imaging databases [36]. Currently, the unfolding of AI in cardiac SPECT/CT and PET/CT imaging has shown utility in three main areas of interest, namely: automation of image detection and segmentation, identification of patients with obstructive coronary artery disease (CAD), and risk assessment of cardiovascular events [37, 38].

Automatic location, reorientation, and segmentation of the left ventricle in SPECT and PET images is achieved with dedicated software able to process both types of nuclear imaging data and has been boosted by the implementation of ML [39]. Such improvements in the automated analysis have demonstrated close correlation with visual scoring of myocardial perfusion images performed by expert readers, supporting their robustness and utility [40].

Both ML and DL methods have been studied to estimate the probability of obstructive CAD. For instance, a single-centre study demonstrated that ML provided with SPECT myocardial perfusion imaging (MPI) and clinical data of 1181 patients showed higher AUC (0.94 ± 0.01) than total perfusion deficit (0.88 ± 0.01) or visual read out, for the detection of obstructive CAD as defined by invasive angiography [31]. ML was also evaluated in the multi-centre REFINE SPECT (REgistry of Fast Myocardial Perfusion Imaging with NExt generation SPECT) registry [41]. The ML algorithm integrating 18 clinical, 9 stress test and 28 imaging variables from 1980 patients showed an AUC of 0.79 [0.77, 0.80], surpassing that of regional *stress* total perfusion deficit (TPD) 0.71 [0.70, 0.73] or *ischemic* TPD 0.72 [0.71, 0.74] in predicting per-vessel chance of early coronary revascularization [34]. In the same registry, DL was utilized in the form of a three-fold feature extraction convolutional layer plus three fully connected layers for analysing SPECT myocardial perfusion raw data and quantitative polar maps of 1638 patients [42]. The output generated a pseudo-probability of CAD per vessel-region and per individual patient and showed a discrete AUC of 0.80 for the detection of ≥ 70% stenosis, that nonetheless outperformed TPD [32]. The DL approach was adapted to a joint analysis of 2-view (upright and supine) data from dedicated cardiac scanners and evaluated in repeated external validation in 1160 patients, improving current perfusion analysis in prediction of obstructive CAD [43]. Such reports have explored the value of clinical and imaging data integration and represent the foundation for further generation of independent systems that provide an automatic interpretation of SPECT and PET images. Important is to realize that the current data needs to be prospectively evaluated/validated in clinical trials.

**Fig. 3:**
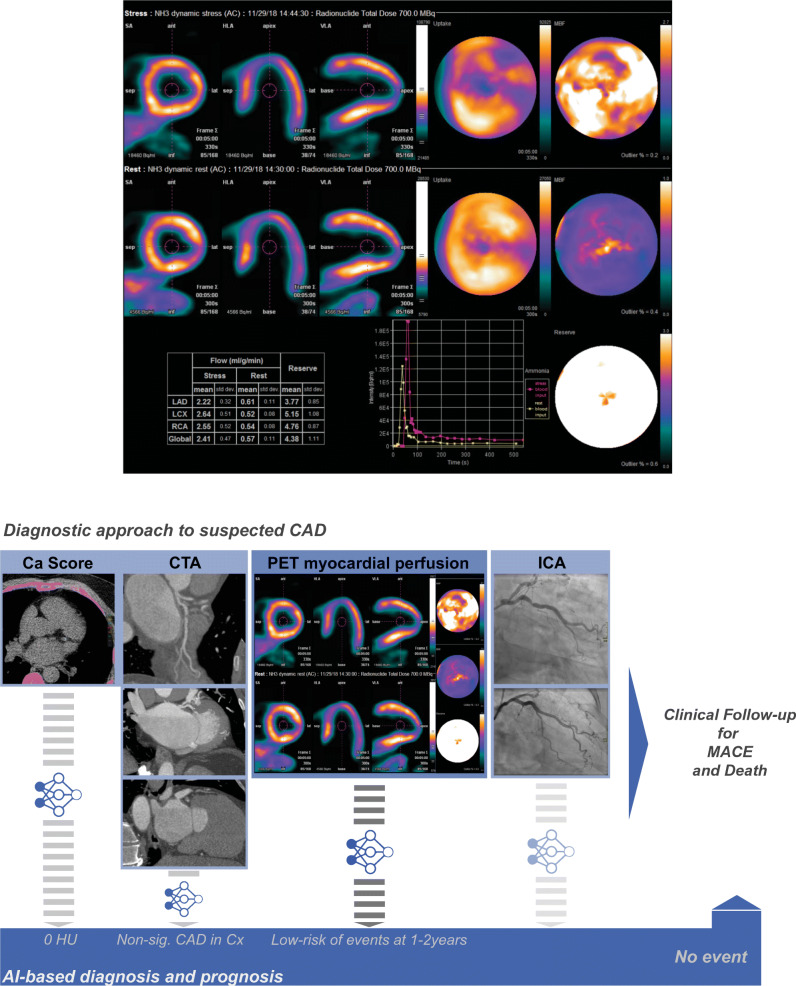
Potential roles of AI in cardiac imaging. Depiction of an exemplary PET/CT case. Male with non-significant atherosclerosis in the left circumflex and overall preserved perfusion reserve in which DL-based processing of PET myocardial blood flow polar maps automatically suggested low-risk of events at a 1–2 years horizon. Transparency on the workflows represents AI implementations that were not used in this particular example, namely automatic calcium score quantification, CTA (FFR) analysis, and ICA analysis. AI*,* artificial intelligence; Ca, calcium; CAD, coronary artery disease; CTA, computed tomography angiography; ICA, invasive coronary angiography; MACE, major adverse cardiovascular events; PET, positron emission tomography

The prognostic value of ML [44, 45] and DL algorithms [27] has also been explored in SPECT and PET imaging of CAD. A large analysis of data considering 28 clinical variables, 17 stress test, and 25 SPECT imaging variables from 2619 patients recently showed the prognostic utility of integrating clinical and imaging-derived numerical data. The study demonstrated a predictive accuracy for 3-year risk of major adverse cardiovascular events (MACE) (AUC = 0.81 [0.78, 0.83]) beyond existing visual or automated perfusion assessments [44]. In a recent study in 20,414 patients, Hu et al. demonstrated the potential application of ML using the XGboost method for the safe cancellation of a rest scan after the stress scan by assigning an AI-based MACE risk score to patients. This approach has demonstrated a much more accurate risk stratification for MACE, allowing 60% of patients to be assigned for stress-only imaging due to their very low risk for MACE, with an annual risk of 1.4%, compared to the visual risk assessment that resulted in 2.1% MACE risk while selecting a similar proportion of patients [45]. Furthermore, in PET imaging, prediction of adverse cardiovascular events has recently been studied through the implementation of *transfer learning*, which allows for data economization while boosting image recognition capabilities and broadening the horizon of network architectures that can be constructed. This was explored in a study evaluating only quantitative PET myocardial perfusion polar maps for the prediction of adverse cardiovascular events at 2 years of follow-up [27]. Notably, the discriminatory capacity of the tailored network (AUC = 0.90 [0.88, 0.92]) even surpassed that of the linear integration of regional myocardial blood flow estimates, clinical and functional variables (see Fig. 3). Of note, the term prediction has been loosely utilized in reports employing binary classification analytics with little weighting of the influence of time in prognostic modelling. As such, current evidence has only demonstrated the retrospective discrimination capabilities of ML algorithms in the identification of patients with a documented adverse event. At this point, it is still unknown whether prospective prediction or prognostic estimations can further outperform existing models and whether clinical actions informed by AI’s prognostic estimates can impact clinical outcomes.

Notably, there is a paucity of data regarding AI implementation in many applications of cardiac nuclear imaging, such as evaluation of endocarditis and infiltrative diseases (amyloidosis and sarcoidosis). This is due to a lack of organized big multicentre datasets suitable for ML analysis. However, such organizational necessities are being addressed and will offer the opportunity to expand the use of AI in these areas. An interesting yet preliminary implementation of AI in this regard will be the cross-generation of cardiac images for advanced alternative imaging (e.g., “pseudo” PET images from MRI data), as documented by Emami et al. [46]. This may offer the opportunity to elucidate whether constitutional MRI data may contain complex dependencies that can translate into, for instance, inflammatory PET findings of practical diagnostic use, but this needs to be evaluated in clinical trials. A particular challenge in any such image generation-based approach is the way the pathological regions will be handled between the different modalities.

In summary, the incorporation of AI in cardiovascular nuclear imaging with SPECT/CT and PET/CT has allowed for refinement in automatic detection and segmentation of raw images. Such exploratory implementations are yet to expand into more complex image analysis such as cardiac FDG-PET images in inflammatory and infiltrative diseases. AI approaches will likely benefit from the integration of multiple clinical, biological, and imaging data to refine the performance of FDG-PET images for the assessment of the diagnostic and risk stratification of patients.

## AI in cardiovascular CT

In cardiovascular CT, AI can be used to identify the presence of disease, to analyse vessels or chambers, and to combine different types of imaging and clinical data to improve diagnosis or prognosis.

Identification of coronary artery calcification (CAC) on non-contrast CT can identify patients with previously unknown CAD. ML can be used to identify and quantify coronary artery calcification [47]. DL has been used to identify CAC on electrocardiogram-gated cardiac CT [48] as well on non-gated CT acquisitions including the heart for other, i.e., non-cardiac indications [49, 50]. van Velzen et al. used DL to identify and quantify calcium on CT using 7240 participants, which included ECG-gated CT, diagnostic CT of the chest, PET attenuation correction CT, radiotherapy planning CT, and low-dose screening CT for lung cancer [51]. The resulting model had an intraclass correlation coefficient of 0.85–0.99 for the identification of CAC, leading to the prospect of routine automated quantification of calcification on thoracic CT. More recently, a study using 20,084 gated and non-gated cardiac CT scans developed a deep learning model to identify coronary calcification with excellent correlation with manual readers (r 0.92, *p* < 0.001) and test-retest stability (intra-class correlation 0.993, *p* < 0.001) [52].

In addition to CAC, other features of cardiovascular disease can also be identified using ML/DL on non-contrast imaging, including the presence of previous myocardial infarction [53], cardiac chamber dimensions [54] or pathologies, and calcification in other vascular beds [50]. Inflammation in the pericoronary adipose tissue can identify patients at increased risk of subsequent cardiac events [55]. DL models can identify and quantify epicardial and thoracic adipose tissue on non-contrast CT [56–58], and this has been shown to better predict major adverse cardiovascular events (MACE) compared to traditional risk factors [59, 60].

Assessment of the coronary arteries is a key element of contrast-enhanced CCTA. Qualitative visual descriptors of stenosis and morphology are limited by observer variability and only describe a portion of the potentially available information present on the images [61]. In order to automatically and quantitatively extract more information, most approaches first require the segmentation of the coronary arteries. Alternatively, ML can be used to identify the coronary centreline and vessel surface, which can then be used for automatic identification of coronary plaque burden [62, 63]. Other authors have used ML directly on imaging data to identify the presence of significant coronary artery disease [64]. A variety of ML techniques have been used to identify calcified and non-calcified plaque and the presence of obstructive CAD on CCTA [65]. To date, these studies are small, and further research is needed. DL has been used to automatically assess coronary artery calcification on CCTA with good accuracy compared to conventional calcium scoring [48].

Alternatively, the identification of CCTA without coronary artery calcification can be used to help prioritize work lists, by identifying scans that can be reviewed less urgently [66]. These techniques can also be used to expand the capabilities of CCTA, for example, to identify lesion-specific ischemia from conventional anatomical images [53]. ML and computational fluid dynamic approaches have been used to assess the hemodynamic significance of coronary artery stenoses by providing CT-fractional flow reserve (FFR) measurements [67–70]. In addition, DL assessment of the left ventricle, without assessing the coronary arteries, has been used to identify patients with functionally significant coronary artery stenoses compared to assessment with invasive fractional flow reserve [71].

Cardiac chamber segmentation on contrast-enhanced CT is a relatively established ML/DL application, with a variety of different techniques employed by different groups [72, 73] Models with more specific tasks have been developed to assist valve implantation [74], electrophysiology assessment of the left atrium [75], and to aid transcatheter aortic valve implantation (TAVI) [76, 77]. A DL model quantifying left atrial volume on non-gated CT showed to be an independent predictor of the presence of atrial fibrillation [78]. Vascular assessment is also possible using similar ML/DL techniques. This includes using DL to segment and measure the thoracic aorta [79] on contrast or non-contrast CT and to identify the presence or risk of acute aortic syndromes [80].

Identification and assessment of radiomic features can be used to expand the analysis of CT beyond what is capable using the naked eye [81]. Radiomic information can expand the capabilities of CCTA by identifying specific imaging markers of vulnerable plaques, such as intravascular ultrasound identified attenuation (AUC: 0.72, CI: 0.65–0.78), optical coherence tomography identified thin cap fibroatheromas (AUC: 0.80, CI: 0.72–0.88) [82], or histological categories (AUC: 0.73 CI: 0.63–0.84) [48]. Even more importantly, radiomics and DL may provide functionalities that significantly increase the capabilities of CCTA, such as identifying PET radionuclide uptake (AUC: 0.87, CI: 0.82–0.91) [82], or deriving calcium-scores from CCTA images automatically [51]. Radiomic analysis of the perivascular fat also holds valuable information and can identify patients who suffer major adverse cardiac events within 5 years of CCTA [83]. Radiomic analysis may also have additive value in differentiating between aetiologies causing prosthetic valve obstruction [84]. In addition, radiomic analysis of the myocardium on non-contrast CT can identify features of myocardial infarction [53].

## CT combined with clinical parameters

ML can also be used to assess a combination of clinical and imaging features to improve prognostic assessment. Models incorporating decision trees are particularly suited for this analysis. Using the CONFIRM (Coronary CT Angiography EvalulatioN For Clinical Outcomes: an InteRnational Multicentre) registry, Motwani et al. showed that a ML-model incorporating clinical and CCTA data outperformed traditional risk scores and CT-derived parameters to predict 5-year all-cause mortality (AUC: 0.79) [84]. Further analysis of this cohort has shown that clinical features and calcium score can be combined in a ML model to predict the likelihood of identifying obstructive disease on CCTA [85], and a ML model incorporating high-risk plaque features further improved the predictive ability [86]. In the MESA (Multi-Ethnic Study of Atherosclerosis) study, a ML model incorporating clinical, biochemical, and imaging biomarkers was superior to cardiovascular risk scores or calcium score alone [87]. The EISNER (Early Identification of Subclinical Atherosclerosis Using Non-Invasive Imaging Research) trial demonstrated using a ML model including clinical findings, coronary artery calcification, and epicardial adipose tissue quantification improved outcome prediction compared to traditional risk scores [59, 88]. Models incorporating explainable machine learning tools are helping to understand the complex interactions of these factors [86]. Future studies should incorporate additional quantitative and qualitative imaging biomarkers in order to optimize prognostic assessment from CT.

## Challenges for artificial intelligence

As with any new technology, it is necessary to identify the advantages of AI, and the associated improvements it may help achieve in terms of image processing and analysis for clinical applications. The field is in the early phase of development (“hype” zone) and as such there is a clear need to identify applications that will have an impact on clinical practice in the short and long term and move forward these indications through an extensive testing and evaluation process. In terms of improving image quality, there is initial evidence that AI-based algorithms do not represent generic solutions to image reconstruction and image analysis tasks and should therefore be trained for specific applications across the different modalities [89]. However, transfer learning and other approaches aiming to minimize the need for the representative training data may alleviate these problems. We may also be able to use transfer learning to gain insights from other domains of imaging, such as oncology research. Larger training datasets allow an increase in the variability of the data based on which the AI learns, and should therefore impact the robustness of the results by reducing the probability of model underfitting and/or overfitting [90]. Furthermore, sharing data between institutions and research groups may allow large steps towards generalization [28]. However, care must be taken to ensure that combined datasets are robust and representative. Sample size calculations for ML analysis, and DL in particular, can be challenging but the number of available events should be considered in the generation of prediction models [91].

In radiomic assessment, multiple factors may influence feature values, including random variations in scanner and patients, image acquisition and reconstruction settings, region of interest segmentation, and image preprocessing [92]. Several studies have proposed to either eliminate unstable features, correct for influencing factors, or harmonize datasets in order to improve the robustness of radiomics [90]. Respiratory motion-induced spatial mismatch between the emission data from PET and the attenuation data estimated from CT can cause moderate to severe artifacts in cardiovascular imaging studies, and severely influence the data quality. Motion correction is warranted to reduce this confounder. Recently published guidelines and checklists aim to improve the quality of future radiomic based AI studies, and transparency has been recognized as the most important factor for reproducibility [90].

Another area of future development concerns the use of explainable AI. This aims to produce models which are less opaque and more understandable for human users. Allowing insight into the decision making process of ML/DL models will facilitate their acceptability by both medical experts and patients. Better understanding of how these models work can help to ensure that they behave appropriately. Furthermore, we can potentially understand more about disease processes by learning how successful ML/DL models work in these areas. One method to do this is to use models which are inherently more understandable and can be interrogated during and after training. Another method is to produce secondary images which combine the underlying radiological image with information from the ML model. These “saliency maps” highlight areas or features in the radiological image which are being used by the ML model. Saliency refers to unique features (pixels, resolution, etc.) of the image in the context of visual processing, and saliency maps are a topographical representation of them [45, 60, 93]. This can aid in the understanding of which aspects of an image are being used by the machine learning model. However, care must be taken with their application as currently available methods do have potential limitations [22].

Ideally, AI algorithms would need to constantly learn and adapt to changes in specific populations or hospital-specific population and scanner hardware and software, allowing for continuous optimization of AI applications [94]. However, the constant evolution and improvement of ML algorithms can challenge existing regulation and approval systems, and new methods for this are being developed. The large discrepancy between the speed of development of AI implementations and that of adequate regulation for safe deployment represents the most important challenge to consider. Ethical codes of conduct have now been established for the development and use of ML in medical imaging and should be adhered to [95].

## Integration in clinical routine

In nuclear cardiology and cardiac CT, AI is trying to find a prominent role in clinical routine beyond the initial steps that have been taken towards automated image processing and analysis. In the currently published data, development and validation of ML in several applications have been demonstrated, including image segmentation [39], measurement of coronary artery calcium scores [48, 50], grading of coronary stenosis on CT angiography [40, 41, 44, 67, 96, 97], and identification of perfusion defects on nuclear imaging scans [40, 41, 44]. Furthermore, ML models based on the integration of clinical and imaging variables have been shown to provide a rapid and precise computation of post-imaging disease or outcome probability [37, 44, 45, 84, 98]. Figure 3 exemplifies such advancing implementations.

However, limited data exist on the actual effects on patient outcomes and costs, and therefore randomized clinical trials are warranted [99]. Prospective validation in representative cohorts and controlled trials are needed to demonstrate the accuracy and efficacy of AI. Efforts are ongoing to collect large databases, including electronic medical records, nuclear or CT imaging datasets, and outcome data for training and validation of AI [35]. Standardization of clinical data recording and imaging protocols as well as efficient dissemination of data will be essential before data from different centers can be used as input by AI.

Although AI offers opportunities to reduce costs, save time, and improve clinical decision making, several practical and ethical aspects have been described that need to be considered in order to integrate AI into the clinical routine [100]. One is the error rate deemed acceptable for an algorithm. Neither man nor machine could be 100% accurate regarding patient risk assessment, but there is an understandable low tolerance for machine errors. Measures to promote the application of AI in clinical practice include appropriate legislation and regulations on the use of AI; transparency in assessment of relevant performance components of AI (with separation of data, performance, and algorithmic transparency and recognition of the uncertainty that can be attributed to an algorithm’s output); robust information technology (IT) governance to handle large amount of data; and training and educational programs on how to appropriately assess and use AI products. The training curricula should promote multidisciplinary collaboration between AI developers, implementers, and clinical practitioners in all relevant fields. In order to promote research on AI, guidelines on how to best construct and apply AI models as well as objectively evaluate their results should be considered.

## Future perspectives

Specific requirements and quality control processes are needed for the development of deep learning-based solutions in medical imaging. Most important to address are concerns regarding the datasets used, the robustness and reproducibility of the proposed solutions, their interpretability, and clinical implementation/acceptability.

### Data sets and harmonization

Large volumes of data are required to test and train ML algorithms, particularly when DL networks are applied on imaging data. High-quality images that are representative of clinical practice are required, and the ground truth should always be based on solid endpoints. Automated annotation, data augmentation, and synthesis can be used to enhance limited datasets but must be used with caution. For testing validation and training, datasets can be split or other methods can be applied, such as k-fold cross-validation [101]. Furthermore, external validation on datasets from separate sites should be performed, to assess expected accuracy of deployed AI in other medical centers. The possibility of using AI for harmonization and standardization of multi-centre imaging studies is gathering momentum and could facilitate the usage of heterogeneous datasets for the training of DL-based algorithms.

### Automation

From data acquisition to disease classification, there are different intervening steps involved (image reconstruction, image segmentation, extraction of imaging biomarkers, image classification, patient stratification) [28]. At the moment, DL-based methods have mostly been developed and tested for each of these individual steps of using medical imaging in clinical practice. One could expect in the future that AI-based algorithms could automatically handle all of these steps in a transparent fashion to the user. Acquired data can be automatically reconstructed using new AI-based algorithms, and it is possible that attenuation correction will be performed using pre-existing CT or MRI data from previous examinations or even completely without the help of morphological image data. Furthermore, it is expected that the segmentation and re-angulation of image data will run automatically. It is also to be expected that, with the help of prognostic models previously developed on large patient groups, a certain risk assessment will be evaluated, e.g., for the presence of a flow limiting coronary artery disease or for the occurrence of MACE. Finally, the incorporation of AI into this envisioned automated workflow (from aquisition and pre-processing to disease and risk identification) is useful for complex cases that will benefit the most from expert clinical analysis in situations of massive data overflow.

### Clinical implementation

With regard to implementation in clinical routine, a certain change in the activities of the medical imaging specialist can be expected. As a first step, tedious and time-consuming work such as data analysis will be performed automatically, and the imaging physician will receive an initial assessment by the system. Deep learning promises to better integrate medical data sources, address the heterogeneity in patient disease types, bridge the gap between omics research and bedside phenotypes, and ultimately enable personalized medicine. Educational programs should be implemented, given the already ubiquitous presence of AI. In medical education, the implementation of a broad AI curriculum is likely to enrich understanding of many conditions in cardiovascular medicine with heterogeneous aetiologies and/or phenotypes. AI also has the potential to utilize data sources to predict the presence of diseases from sources which we currently do not consider as relevant information, such as facial photos [102]. More AI application is needed for the growing field in cardiovascular infection, inflammation, as described recently in the procedural recommendations of cardiac PET/CT imaging [103]. The use of AI to optimize cardiac PET/MRI is being developed in this relatively new imaging modality [104]. In particular, the use of DL to create pseudo-CT images to improve PET/MRI attenuation correction is under active development [104, 105]. An important challenge for PET/MRI is that MRI sequences for attenuation correction do not provide a complete linear scale, as is available for PET/CT attenuation correction. The use of DL to transform MR and/or PET images into pseudo-CT images that could be used for attenuation correction would therefore improve the accuracy of PET/MRI [104].

### Ethical issues

There are a number of ethical issues which should be considered at all stages of development and use of AI for medical imaging. Firstly, the data used to train the AI models should be used with adherence to local and national policies which consider aspects such as informed consent, privacy, data protection, and data ownership. The use of patient information without informed consent may be possible if certain criteria are met, such as the lawful basis under General Data Protection Regulation, EU 2016/679 (GDPR) or national regulations. However, it is important that patient privacy is maintained, and robust de-identification and anonymization of medical images are required. This includes both imaging meta-data and potentially identifiable features in the images themselves. Efforts should also be made to ensure the data is representative in order to avoid the impact of selection and other biases on the AI algorithm and to ensure generalisability. Secondly, transparency in how the AI algorithm is trained and functions is required, and “black box” algorithms should be discouraged. Thirdly, for clinical applications, the accuracy and safety of the AI algorithms should continue to be monitored, and interpretation by a trained clinician will remain important. Legal and liability issues will vary between countries. There are also potential threats from malicious attacks both during training and use of AI algorithms, and therefore attention to cyber security vulnerabilities is important. Ethical codes for AI research and the use of AI in clinical practice have been established [95], and it is also important that AI research adheres to suitable reporting standards [106].

In conclusion, the application of AI in the cardiovascular field is bringing new possibilities in early detection and diagnosis of CVD, better clinical decisions, outcome prediction, or prognosis evaluation. The finding of the appropriate balance between fully autonomous AI and physician supervision is a new and major challenge. If AI algorithms are at least as accurate and reproducible as assessment by physicians in a dedicated task, it may help in the daily practice by improving patient management. On the other hand, the medical community is not ready, nor should it be, to follow blindly “black box” algorithms, and several key elements for future application of AI in cardiovascular imaging need to be clarified (Table 1). Further discussion of the ethical and legal issues are required before AI shapes the medical practice of the future. Explaining the features behind AI prediction will be central for the physician’s ability to interact and use AI-based systems. Within this context, the development and application of AI-algorithms will be very much welcomed. Finally, guidelines must be developed to standardize broad applications of AI in medicine.

**Table 1:** Key elements for the future: advantages, challenges, and solutions of AI in cardiovascular hybrid nuclear medicine and CT imaging

Advantages	Challenges	Solutions
Precision-, accuracy-, and data-driven decisions for optimal diagnosis and monitoring treatment	Complexity and costs	Improvements in hardware and software
Improve inter-/intra-observer reproducibility	“Black box”: limited/lack of interpretability	Development of user-friendly software solutions to facilitate AI research among clinicians
Time-saving	Privacy, security, and ethical issues	Creating local, national, and international ethical guidelines
Second reader assistance	Regulation, legal, and liability issues	Creation of multi-national available medical data registries
Integration of large and diverse data	Integration of expert and machine decision making	Providing developed AI algorithms as open source and multicentre collaborations
	Changes in job descriptions	Life-long learning
	Availability of large and diverse data sets and limited data in inflammatory and infectious cardiovascular diseases	Creation of multi-national available diverse data sets, including clinical data

## Declarations

### Liability statement

This position paper summarizes the views of the EANM Cardiovascular Committee, Infection & Inflammation Committee, and Physics Committee, and the EACVI. It reflects views for which the EANM and the EACVI cannot be held responsible. This position paper should be taken into context of good practice of nuclear medicine and do not substitute for national and international legal or regulatory provisions.
